# Cardiac Light-Sheet Fluorescent Microscopy for Multi-Scale and Rapid Imaging of Architecture and Function

**DOI:** 10.1038/srep22489

**Published:** 2016-03-03

**Authors:** Peng Fei, Juhyun Lee, René R. Sevag Packard, Konstantina-Ioanna Sereti, Hao Xu, Jianguo Ma, Yichen Ding, Hanul Kang, Harrison Chen, Kevin Sung, Rajan Kulkarni, Reza Ardehali, C.-C. Jay Kuo, Xiaolei Xu, Chih-Ming Ho, Tzung K. Hsiai

**Affiliations:** 1 School of Optical and Electronic Information, Huazhong University of Science and Technology, Wuhan, China; 2 Department of Mechanical & Aerospace Engineering, University of California, Los Angeles (UCLA), Los Angeles, CA, USA; 3 Department of Bioengineering, UCLA, Los Angeles, CA, USA; 4 Department of Molecular, Cellular and Integrative Physiology, UCLA, Los Angeles, CA, USA; 5 Division of Cardiology, Department of Medicine, UCLA, Los Angeles, CA; 6 Department of Electrical Engineering, University of Southern California, Los Angeles, CA, USA; 7 Division of Cardiology, Veterans Affairs Greater Los Angeles Healthcare System, Los Angeles, CA, USA; 8 Department of Biochemistry and Molecular Biology, Mayo Clinic, Rochester, MN, USA.

## Abstract

Light Sheet Fluorescence Microscopy (LSFM) enables multi-dimensional and multi-scale imaging via illuminating specimens with a separate thin sheet of laser. It allows rapid plane illumination for reduced photo-damage and superior axial resolution and contrast. We hereby demonstrate cardiac LSFM (c-LSFM) imaging to assess the functional architecture of zebrafish embryos with a retrospective cardiac synchronization algorithm for four-dimensional reconstruction (3-D space + time). By combining our approach with tissue clearing techniques, we reveal the entire cardiac structures and hypertrabeculation of adult zebrafish hearts in response to doxorubicin treatment. By integrating the resolution enhancement technique with c-LSFM to increase the resolving power under a large field-of-view, we demonstrate the use of low power objective to resolve the entire architecture of large-scale neonatal mouse hearts, revealing the helical orientation of individual myocardial fibers. Therefore, our c-LSFM imaging approach provides multi-scale visualization of architecture and function to drive cardiovascular research with translational implication in congenital heart diseases.

The advent of three-dimensional imaging of biological organisms and tissues provides a paradigm shift to interface optical imaging with cardiovascular research. Unraveling cardiac morphogenesis, regeneration, differentiation and proliferation require deep tissue penetration to visualize the dynamic events with high spatiotemporal and depth resolution. While modern light microscopy techniques, such as wide-field microscopy and confocal scanning microscopy, have enabled spatial resolution to image intracellular organelles, insufficient axial resolution and noticeable photo-damage remain a challenge. The 3-D post-imaging reconstruction and stitching for large sample size renders the imaging processes laborious and prolonged^1^,^2^,^3^. The advent of Optical Coherence Tomography (OCT) and Optical Projection Tomography (OPT) has enabled 3-D and non-invasive imaging of large specimens. OCT allows imaging of living samples by light sectioning through coherence^4^,^5^ and OPT operates on a similar principle with X-ray computed tomography to image chick embryos with intermediate sizes at high resolution. However, OCT is limited from fluorescent imaging due to the lack of coherence, and OPT is limited by its spatial resolution and recording rate^6^. Recently, the use of micro-Computed Tomography (CT) with intrinsic resolution limit (15–45 μm) has provided high spatial and temporal resolution for imaging newborn (<3.5 mm) and fetal mouse hearts (2 mm) with routine operation (<35 min) for congenital heart disease^7^. In addition, micro–Magnetic Resonance Image (MRI) has allowed for live imaging of mouse brain with high spatial resolution (30 × 30 × 60 μm) and compatible scanning acquisition time (<45 min)^8^. Both micro-CT and micro–MRI are limited from tracking the fluorescently labeled live zebrafish and neonatal hearts for cardiac development, injury and repair. In this context, Light Sheet Fluorescence Microscopy is an emerging imaging modality for multi-dimensional, -scale, and -channel visualization of cardiac architecture and physiology accompanied by rapid and precise tracking of multi-fluorescently labeled intracellular, cellular or tissue components from several microns to millimeters^9^,^10^,^11^.

Light Sheet Fluorescence Microscopy (LSFM) has allowed for high speed, precise tracking of multi-fluorescently labeled cells or tissues of interests within a complex and dynamic cardiovascular environment^10^,^11^. Unlike conventional wide-field and laser scanning microscopy, LSFM applies two separate optical paths for plane illumination and fluorescence detection. Instead of wide-field excitation or point scanning in which the excitation path is parallel to the detection path, LSFM selectively illuminates an ultra-thin plane of the sample via a sheet of light orthogonal to the detection path. By providing sharp and in-focus excitation along the axial direction, LSFM reduces the photon burden to the sample, enhances the image contrast via eliminating out-of-focus contamination, and improves the axial resolution under a large field-of-view. With its numerous structure variants, including Selective Plane Illumination Microscopy (SPIM), Multidirectional SPIM (m-SPIM), Digitally Scanned Light Sheet Microscopy (DSLM), Objective-Coupled Planar Microscopy (OCPI), Oblique Plane Microscope (OPM), and Bessel-Beam-based Light Sheet Microscopy, being widely developed in recent years, LSFM is on the verge of becoming a predominant visualization technique for a broad range of life science research^12^,^13^,^14^,^15^,^16^. Ahrens *et al.* demonstrated functional imaging of neurons at nearly one-second resolution^17^. Schmid *et al.* further measured endothelial cell migration patterns and tissue remodeling in the early endoderm^18^. LSFM is uniquely powerful for multi-scale imaging to unravel developmental milestones over a dynamic range of length and time scales.

Here, we developed a cardiac LSFM (c-LSFM) system to image various cardiac architectures ranging from hundreds of microns to several millimeters. To image rapid cardiac contraction from a large region-of-interest (ROI), we integrated several optimization algorithms with the c-LSFM system to enhance temporal and spatial resolution. When imaging a contracting embryonic zebrafish heart, the 2-D plane images obtained from the conventional LSFM significantly improved demarcation of the cardiac boarders for computational fluid dynamics and myocardial strain studying **(Video S1)**. However, we further enhanced the temporal resolution to reconstruct the rapidly contracting 4-D embryonic zebrafish hearts (3-D spatial and time domain) by integrating a retrospective gating algorithm with the c-LSFM system. Next, we introduced a resolution-enhancement technique to offset the decreased spatial resolution from the low numerical aperture (N.A.) and low magnification of the detection lenses; thereby, allowing for imaging macro-scale neonatal mouse hearts with high spatial resolution under a large field-of-view (FOV). We further achieved cellular resolution to uncover the helical orientation of individual myocardial fibers, as well as the bi- and tri-cuspid valves, muscular ridges and trabecular network. In this context, we have built on the established SPIM principle for tunable c-LSFM imaging to uncover cardiac architecture, development and remodeling otherwise challenging with existing imaging modalities. We introduced a widely tunable c-LSFM system for high-speed, large-FOV, and long-term multi-scale imaging ranging from zebrafish model to the higher vertebrate with translational implication to studying congenital heart diseases in the mouse models.

## Results

### Implementation of Cardiac Light-Sheet Fluorescence Microscopy (c-LSFM)

**Figure 1:**
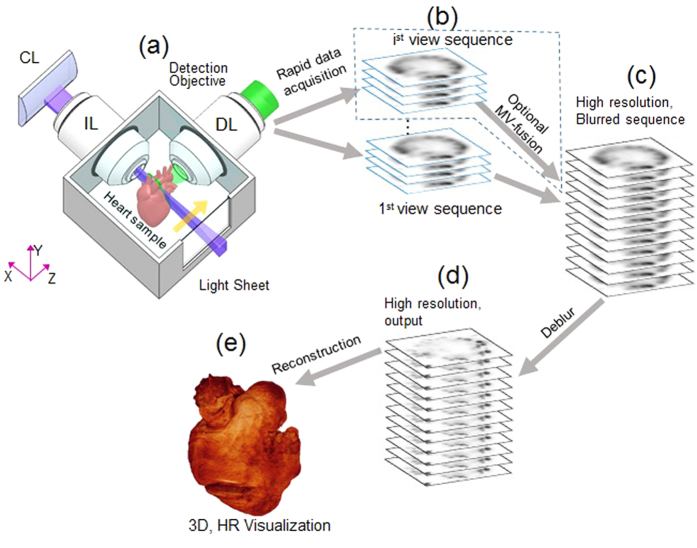
Implementation of cardiac Light-Sheet Fluorescence Microscopy (LSFM). (**a**) The optical setting of LSFM. A laser beam (purple) is collected and focused by the beam expander to optimize the beam size. A cylindrical lens (CL) converts the laser beam to a sheet of laser light to illuminate a thin layer of the sample. The sample is mounted at the intersection of the illumination lens (IL) and detection lens (DL). The illuminated 2-D layer (fluorescent detection in green) is captured by the high-frame rate CMOS camera. The illumination axis is orthogonal to the detection axis, and the illumination optics is designed to illuminate a very thin volume around the focal plane of the detection objective. The configurations of light-sheet illumination and fluorescence detection are highly tunable to accommodate for various heart samples. (**b**) The plane fluorescent images at different axial (z) depths are sequentially captured by the camera (**c**,**d**). For non-transparent fetal mouse hearts, multi-view (MV) techniques are applied to rotate the samples for multi-view imaging, followed by registering and fusing these views into a 3-D cardiac architecture. An iterative deconvolution technique is applied to the blurred sequence for high resolution. (**e**) A digitally reconstructed heart is accomplished by stacking the deconvolved images.

The workflow of c-LSFM was characterized by the orthogonal optical paths and multi-dimensional reconstruction of multi-scale cardiac structures with high resolution (Fig. 1). The optical setting is based on Selective Plane Illumination Microscopy (SPIM)^19^. In the plane illumination path, the combination of a cylindrical lens together with an objective line focused the collimated beam into a hyperbolic light sheet with tunable parameters for illuminating cardiac structures (Fig. 1a). The orthogonal detection path collected the correspondingly excited fluorescence signals under various magnifications ranging from 2X to 20X. To cater the need of frequent switch of detection objectives for various sizes of cardiac samples, we use the long working distance air detection objective combined with post resolution enhancement to replace the water sealing design in original SPIM and improve the convenience of operation. When the heart scanned through the light sheet along the z direction, the sCMOS camera (Hamamatsu, ORCA flash 4.0) located at the terminal end of the detection path simultaneously recorded a stack of 2-D plane images along different z depths (Fig. 1b). At each z depth, only the thinly illuminated plane emitted fluorescence; thus, the captured frames were free of out-of-focus excitation and had high axial resolution. The plane illumination mode and high-frame rate of sCMOS camera allowed completion of the entire 3-D scanning and data acquisition within a few minutes. While the single view scanning was commonly applied for the vast majority of our cardiac specimens, multiple view scanning was an alternative to image the high-scattering cardiac tissues. The multi-view fusion technique restored the entire cardiac architecture from the computationally acquired multi-view dataset^20^. Finally, a spline interpolation and an iterative 3-D deconvolution were subsequently applied to the reconstructed image stack to compensate for the under-sampling of camera and remove the image blurs (Fig. 1c,d). As a result, a 3-D “digital heart” was reconstructed in the last step to provide visualized output with high spatiotemporal resolution and high dynamic range.

### Calibration of light-sheets for multi-scale imaging

**Figure 2:**
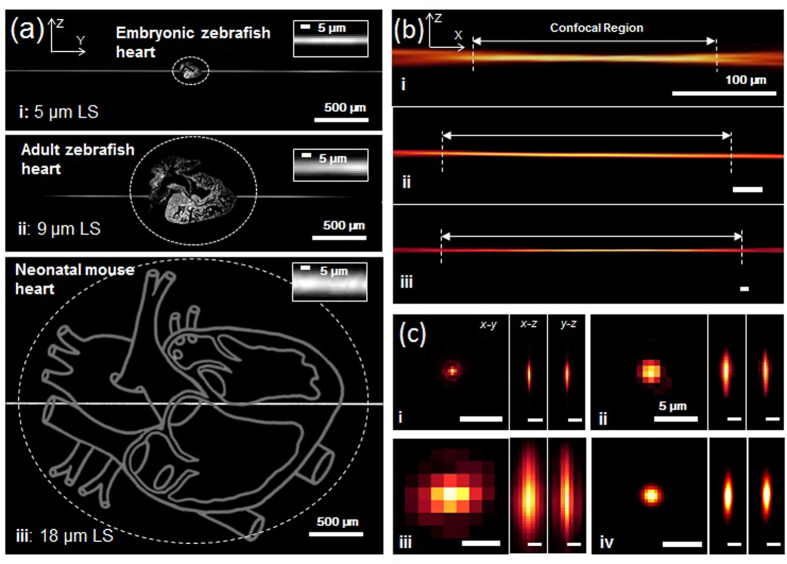
Light-sheet profiles for reconstructing cardiac architecture. (**a**) The axial confinement of the light-sheet (LS) was used for sectioning the (i) embryonic zebrafish, (ii) adult zebrafish, and (iii) neonatal mouse hearts. The small aperture of the slit reduced the beam width to render the waist of laser sheet less focused and wider (shown in the inserts). LS: light sheets. (**b**) The changes in Rayleigh Range corresponded to the area available for light-sheet sectioning. The double-headed arrow line indicates the Rayleigh range (confocal region), in which the light-sheet is considered to be uniform. The scale bars are 100 μm in length for the sub-images in (i), (ii) and (iii). (**c**) Imaging a 400 nm fluorescent bead (sub-resolution point source) was compared with the (i) 5μm light-sheet (LS) detected by the 20X/0.5 detection objective (DO), (ii) 9 μm LS by 10X/0.3 DO, (iii) 18 μm LS by 4X/0.13 DO and (iv) 18 μm LS by 4X/0.13 DO, with resolution enhancement applied. The FWHM extent of image blurring from the point source in the x-y, x-z and y-z plane reflects the lateral and axial resolution.

In our c-LSFM system, the light-sheet profile was widely tunable for multiple-scale cardiac samples. Typically, three light-sheet configurations were generated to illuminate the embryonic zebrafish heart (100–150 μm), adult zebrafish heart (500–1500 μm), and neo-natal mouse heart (3000–5000 μm) (Fig. 2a). To provide a uniform plane illumination across the entire sample, the confocal region of the light sheet was finely tuned to cover the sample’s transverse dimension (Figs 2b and S1). Within a certain size of lateral confocal region, the axial extent of axial resolution of the light sheet was also determined by the property of Gaussian beam (Fig. 2a). To characterize the generated light sheets, we used a monochrome CCD profiler to sequentially acquire the projections of the light sheets along their propagation. The extent of axial projection was directly imaged at the waist of the light sheet by the profiler, and the confocal range was further reconstructed by stacking the projections. The thickness of the light sheet, defined as the axial full width at half maximum (FWHM) value of the beam waist, was measured at ~5 μm for the embryonic zebrafish heart (i), ~9 μm for the adult zebrafish heart (ii), and ~18 μm for the neo-natal mouse heart (iii) (Fig. 2a). The lateral confocal ranges with respect to these three axial extents were profiled (Fig. 2b, i–iii). The detection objectives were 20X/0.5 for the embryonic zebrafish heart, 10X/0.3 for the adult zebrafish heart, and 4X/0.13 for the neonatal mouse heart to capture the full region-of-interest (ROI). Once the thickness of the light-sheet for excitation and the objective lens for detection were determined, we obtained the lateral and axial resolution for each configuration by measuring the point spread function (PSF). We imaged the fluorescent point source (polystyrene beads, average size ~400 nm) by applying the aforementioned 3 light-sheet configurations, and demonstrated the lateral and axial resolution of the c-LSFM system for the individual configurations by measuring the FWHMs from x-y, x-z, and y-z plane images (Fig. 2c). Under the macro configuration, we further compared the results before and after the resolution enhancement was applied (Fig. 2c, III and IV). The resolved FWHMs of the point source were reduced from ~4.5 μm to ~2 μm laterally and from ~18 μm to ~10 μm axially.

### Functional analyses of zebrafish embryos

**Figure 3:**
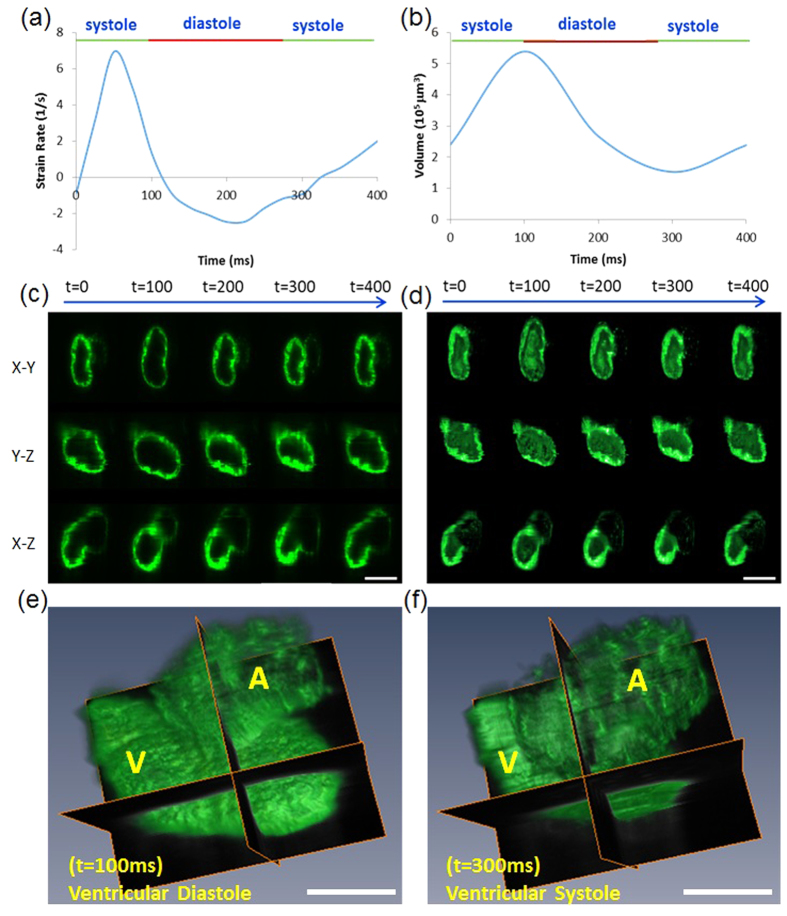
4-D synchronized images to quantify global longitudinal strain rates and volume change of the ventricle at 100 hours post fertilization (hpf). (**a**) Changes in global longitudinal strain rates were quantified during the entire cardiac cycle. (**b**) The ventricular volume was measured in terms of EDV at 95.4 × 10^5^ μm^3^ and ESV at 1.5 × 10^5^ μm^3^, respectively. (**c**) LSFM images captured the zebrafish hearts in the x-y, x-z, and y-z planes during the cardiac cycle. (**d**) 4-D synchronized LSFM-acquired images revealed endocardial trabeculation in the x-y, x-z and y-z plane during the cardiac cycle. (**e,f**) 4-D zebrafish cardiac motion was captured during ventricular diastole and systole. A: Atrium, V: Ventricle. Scale bar: 50 μm

The global longitudinal strain rate is defined as the shortening of a defined global longitudinal length over time. We measured the strain rates at 100 hours post fertilization (hpf) over the cardiac cycle period (Fig. 3a). We applied high frame-rate acquisition and acquired multiple image sequences to improve the spatial and temporal resolution in response to variable cardiac cycles^10^ (**Video S2,S3**). The 4-D synchronized LSFM post-image processing allowed analysis of the instantaneous changes in ventricular volume in living zebrafish embryos in the x-y, y-z, and x-z planes (Figs 3b, S2 and Video S4), from which we quantified the mean stroke volume (4.1 × 10^5^ μm^3^) and ejection fraction (74.5%) (Table S1). At 100 hpf, we revealed the prominent trabecular network that provides oxygenation and nutrition to the myocardium to enhance cardiac contractile function^21^.

### Doxorubicin-induced cardiac injury in adult zebrafish

**Figure 4:**
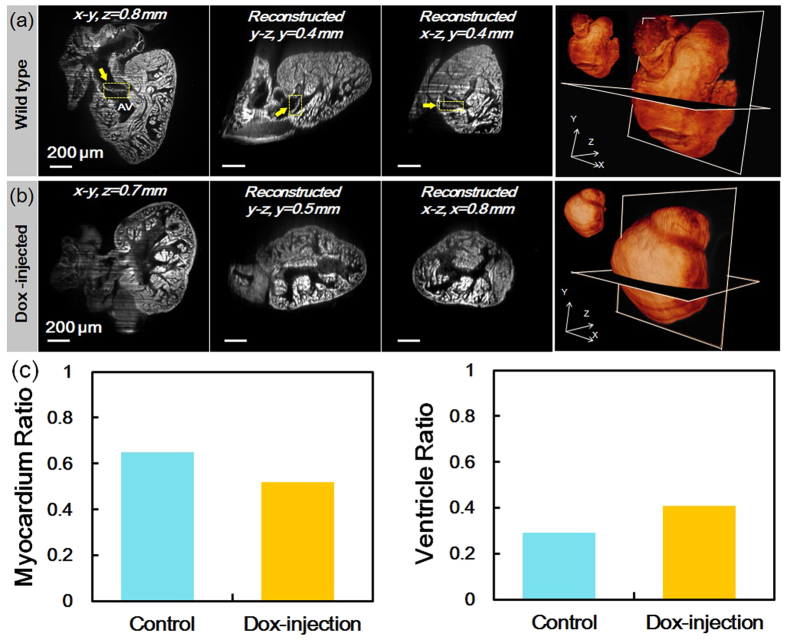
Rapid 3-D images to recapitulate trabeculated network in response to doxorubicin (Dox) treatment in the adult zebrafish. (**a**) A representative wild-type zebrafish heart at 120 dpf. The coronal, sagittal and transverse planes of the heart displayed a compact trabecular network. The atrioventricular valve (AV) was identified (yellow arrows). Scale bars are 200 μm in length. In the rightmost column, a 3-D rendering of the “digital heart” was reconstructed by stacking 500 slices of plane images in volume. The 3-D structure of the “digital heart” can be assessed by arbitrary cropping. (**b**) A representative Dox-injected zebrafish heart at 120 dpf. The endocardial cavity appeared enlarged and the trabecular network was accentuated. (**c**) The quantified volume ratios of the myocardium (left) and the ventricle cavity (right) in the whole heart.

By integrating tissue clearing technique with c-LSFM, we were able to implement rapid 3-D imaging of the intact adult zebrafish hearts without cryostat sectioning. The fast BABB (1:2 benzyl alcohol:benzyl benzoate mixture) clearing technique rendered the entire hearts translucent with preservation of the fluorescently-labeled tissues of interest. The combination of sustained fluorescent signal from the transgenic *Tg*(*cmlc:gfp*) green fluorescent protein–labeled cardiomyocyte light-chain (cmlc) and the high detection efficiency of the c-LSFM system allowed for rapid light-sheet scanning of the entire hearts within 1 minute, followed by high resolution 3-D reconstruction using Amira visualization software. We demonstrated the coronal, sagittal, and transverse plane images of a wild-type heart at 120 days post fertilization (dpf), revealing the trabecular network and atrioventricular valve (AV) (Fig. 4a). The volumetric rendering of the heart was achieved by stacking 500 z-slices together, allowing for 3-D visualization and quantification of functional phenotypes from various image views (Fig. 4a). Prior to doxorubicin (DOX) injection, the trabecular network appeared compact in association with a small ventricular cavity. Following chemotherapy injection at 90 dpf, adult zebrafish developed an accentuated trabecular network and an enlarged ventricle at 120 dpf in comparison with the wild-type (Fig. 4b). We further validated these findings by quantifying the occupancy ratio of the myocardium to the entire heart as ~65% in wild-type fish versus ~52% in DOX-injected fish (Fig. 4c). As a corollary, the volume ratio of the ventricle cavity was ~27% versus ~41% (Fig. 4d). These findings suggest that the c-LSFM system unravels hypertrabeculation as a new phenomenon of myocardial response to injury and repair.

### Cardiac LSFM (c-LSFM) to image neonatal mouse architecture

**Figure 5:**
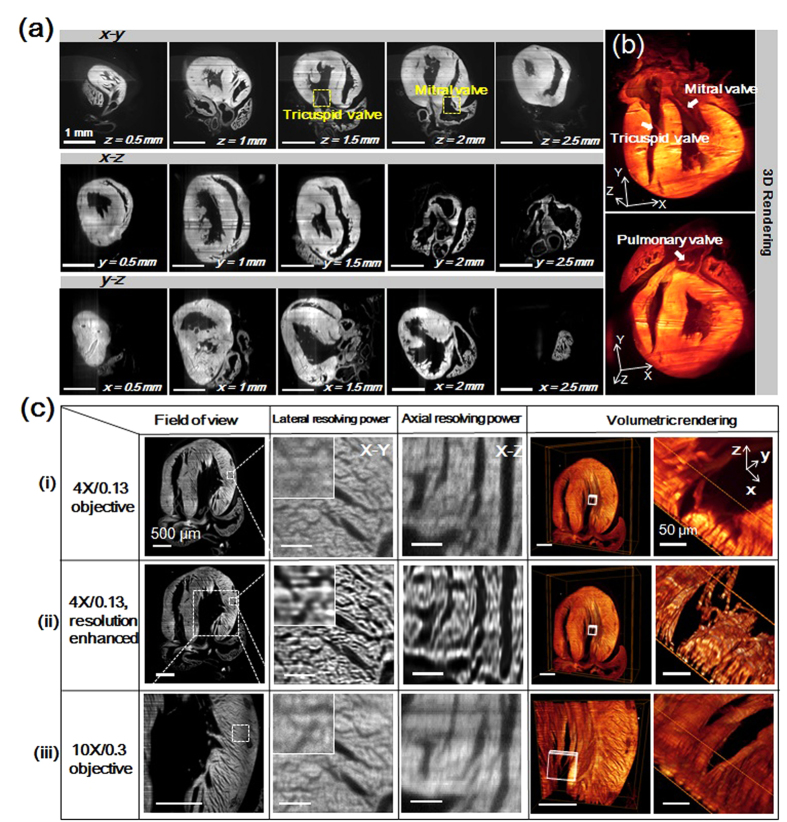
Cardiac LSFM (c-LSFM) imaging of a 1-day neonate mouse heart with enhanced cellular resolution. (**a**) The coronal, sagittal, and transverse planes at different depths uncover 3-D architecture. Scale bars are 1 mm in length in all of the sub-graphs. (**b**) The boxes were cropped from the volume rendering of the reconstructed “digital heart” to reveal the endocardial architecture. (**c**) The cardiac architecture is compared with the (i) 18 μm light-sheet and 4X/0.13 objective, (ii) 4X/0.13 resolution enhanced images, and (iii) 9 μm light-sheet and 10X/0.3 objective. Magnification from left to right reveals the field of view, lateral, and axial resolving power, followed by the volumetric rendering effects of 3 configurations. Myocardial orientation was resolved in detail in the resolution-enhanced c-LSFM group. All scale bars are 500 μm, except for 50 μm in the rightmost column.

We further calibrated the light-sheet configuration to image the neonatal mouse heart with a size in the range of several millimeters. A 15 μm light-sheet sectioning plus a 4X/0.13 detection lens were applied to image the entire sample with a large FOV. By stacking the recorded z slices into a volume (600 slices with 6 μm in the step size), we demonstrated the reconstructed sagittal and transverse planes, as well as the original coronal planes, to reveal the 4-chamber cardiac structures (Fig. 5a). The volumetric renderings of the reconstructed “digital heart” further revealed the 3-D morphology with high axial and spatial resolution. By cropping and rotating the 3-D heart, we highlighted the bicuspid, tricuspid, and pulmonic valves, the trabecular network in the atrial appendages, as well as the myocardial fiber orientation (Fig. 5b,c and Video S5).

**Figure 6:**
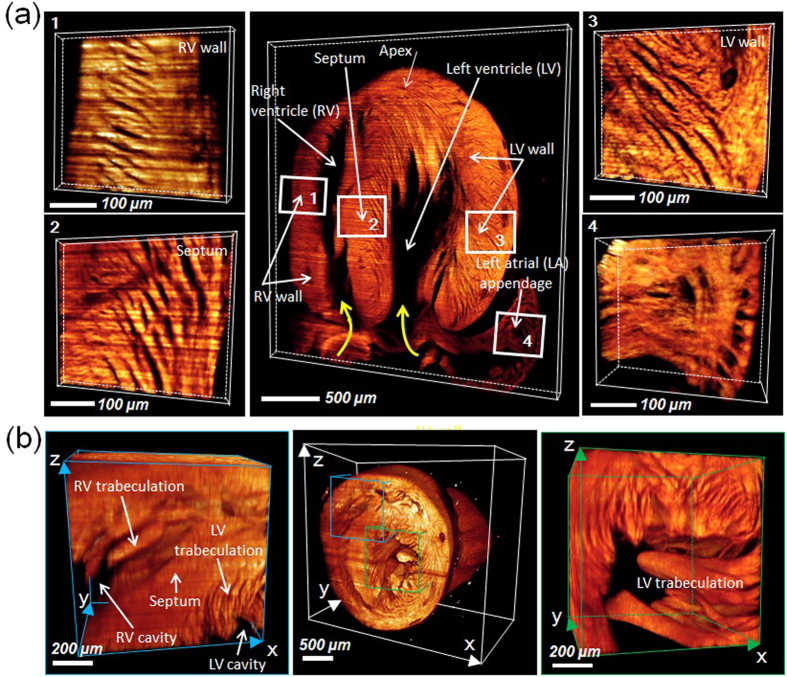
High resolution architecture of neonatal mouse hearts. (**a**) 3-D LSFM revealed the distinct helical organization of individual cardiomyocyte fibers from the right ventricular wall to septum to left ventricular walls (zones 1, 2, and 3), providing insights into the mechanics of ventricular contraction in RV vs. LV. Endocardial structure of the left atrial appendage revealed the muscular ridge and muscular trabeculation (zone 4). The yellow curved arrows indicate the orientation of cardiomyocyte fibers. (**b**) Ultrastructure in the RV (zone 1) and LV cavity (zone 2) unravel trabeculation/papillary muscle (zone 1). LV: left ventricle; RV: right ventricle; LA: left atrium.

However, the space-bandwidth product of our c-LSFM system was limited by the use of a low power objective lens (4X/0.13) and relatively raw voxel sampling under large FOV (1.625 × 1.625 × 8 μm in case of normal sampling). Thus, the lateral and axial resolution prior to post-imaging processing appeared insufficient to resolve the cellular details, such as a single myocardial fiber under a large FOV. For this reason, we implemented deep over-scanning of the sample and applied a resolution enhancement processing based on the over-scanned data to increase the space band-width product. First, to reduce the information loss from under-sampling as much as possible, we ran an over-scanning of 3 μm step size (being 6 times smaller than the light sheet thickness) in the axial direction during image acquisition and scaled up the obtained 3-D image 3 times at lateral direction using b-spline interpolation. Then, we deconvolved the pre-processed 3-D image with measured 3-D point-spread-function of the optical system (interpolated to the same voxel spacing with the image data), to further recover the image from blurring and substantially enhance the resolution. The iterative 3-D deconvolution conducted in this work was run on the open source ImageJ platform using its “parallel iterative deconvolution” plugin, “3-D iterative deconvolution” module. We selected the MRNSD (Modified Residual Norm Steepest Descent) or WPL (Wiener Filter Preconditioned Landweber) option, which are both non-negatively constrained algorithms, with appropriate preconditioning parameters to solve the final resolution-enhanced output (Fig. S3). During computation, we set the max number of iteration (e.g., 50) and mean delta threshold (e.g., 0.01) to determine the convergence. In most cases, the 3-D deconvolution could be completed within 20 iterations and generated the final deblurred output. In Fig. 6c, the top panels (i) show the raw c-LSFM imaging with blurred cellular structure. The middle panels reveal enhanced resolution to map the cardiac architecture at the cellular resolution. Upon zooming into the left ventricular wall, the volumetric rendering unraveled the myocardial fibers with diameter <10 μm. In the lower panels, images illuminated by the 9 μm light-sheet and detected by the 10X/0.3 objective lens were compared with the upper and middle panels. This comparison demonstrated the super-resolved cardiac architecture with the use of a lower magnification lens to achieve a resolving power superior to those of higher magnification. We further demonstrated this enhanced imaging capability to reveal the distinct helical orientation of cardiomyocyte fibers in the ventricular and septal walls, as well as the muscular ridges and trabeculation in the left atrial appendage (Fig. 6 and Video S6). Overall, by integrating our c-LSFM with resolution-enhancement computation, we decoupled limited resolution from the large FOV and achieved cellular resolving power over a meso-scale specimen.

## Discussion

The main contribution of our in-house c-LSFM system lies in its multi-scale and rapid cardiac imaging with high axial and temporal resolution to uncover cardiac developmental structures in both zebrafish embryos and neonatal mice as well as revealing ventricular changes in response to chemotherapy-induced cardiotoxicity. The application of optical clearing of the adult cardiac samples for c-LSFM, followed by the post-enhancement algorithms, revealed 3-D hypertrabeculation in response to doxorubicin-induced injury in zebrafish hearts (hundreds of microns) and valvular structures and helical orientation of cardiomyocyte fibers in the ventricular walls of the neonatal mouse hearts (a few millimeters) (Fig. S4). We quantified the time-dependent contractile function of zebrafish embryos at 100 hpf. We visualized the neonatal cardiac architecture by using a low power magnification to resolve cellular structures otherwise challenging with existing micro-CT imaging modalities^7^. We distinguished bicuspid from tricuspid valves, and unraveled trabecular networks; thereby, providing anatomical, functional, and pathophysiological phenotypes to drive the future investigation of cardiac injury, repair, and development.

Unlike the conventional Selective Plane Illumination Microscopy (SPIM), our c-LSFM system was designed to accommodate multi-scale cardiac samples^22^,^23^. SPIM requires the illumination and detection lenses being sealed into a water chamber in which the samples are in close alignment with a short working distance; whereas our c-LSFM system applies a long working distance and non-water dipping objectives. In addition, the large working space allows for scanning of entire macro-size samples, and the integration of the resolution enhancement technique decouples the requirements for high numerical aperture (N.A.), water-dipping objectives for high resolution, and the large space-bandwidth product. Moreover, avoiding the need to seal the objectives in the water chamber enables frequent changes of objectives for various sample conditions and dimensions. Based on the optical principle of SPIM, we have developed a tunable c-LSFM system for multi-scale and rapid cardiac light-sheet imaging.

When imaging a large sized cardiac structure, such as the neonate mouse heart, the raw LSFM data obtained under a low magnification and a large field-of-view setting were subject to significant image blurring caused by (1) limited optical power (the small N.A. of the detection objective and thick illuminating light-sheet), and (2) insufficient digital readout (under-sampling from the camera). This image blurring generated an unsatisfactory resolution to distinguish the fine cellular structures. To address this issue, we implemented a step-by-step image processing to recover the 3-D sacrificed spatial resolution over the entire volume-of-view. To reduce the information loss from under-sampling, we performed 4 to 8 times over-scanning (the step size is 4–8 times smaller than the light-sheet thickness) along the axial direction during image acquisition, followed by scaling up the obtained 3-D image by 2- to 4-times at lateral direction using b-spline interpolation. To further de-blur the image and to substantially enhance the resolution, we deconvolved the pre-processed 3-D image with the measured 3-D point-spread-function of the optical system (that was interpolated to the same voxel spacing with the image data). The iterative 3-D deconvolution was run on the ImageJ platform using its “parallel iterative deconvolution” plugin, and “3-D iterative deconvolution” module. We chose the MRNSD (Modified Residual Norm Steepest Descent) or WPL (Wiener Filter Preconditioned Landweber) option, both of which are non-negatively constrained algorithms, with appropriate preconditioning parameters to solve the final resolution-enhanced output. During computation, we set the max number of iteration (e.g., 50) and mean delta threshold (e.g., 0.01) to determine the convergence. In most cases, the 3-D deconvolution could be completed within 20 iterations. We further provided image dataset prior to and post 4-D synchronization algorithm. Prior to synchronization, image dataset from Z_1_, Z_2_ Z_3_, and Z_4_ at same time point are in a different cardiac cycle. However, post 4-D synchronization, Z_1_, Z_2_ Z_3_, and Z_4_ are synchronized at same time point (Fig. S5). Therefore, 3-D images at each time point permitted analysis of cardiac mechanics based on time-dependent volume changes (Table S1).

While the 4-D synchronized beating zebrafish heart was reported (Mickoleit, *et al.* “High-resolution reconstruction of the beating zebrafish heart.” *Nature Methods* (2014)), we performed additional 4-D functional analysis of cardiac mechanics by time-dependent 3-D volume change based on our resolution enhancement algorithm; thereby, uncovering 4-D *in vivo* physiological parameters, including end-diastolic volume, end-systolic volume, stoke volume, ventricular ejection fraction, heart rate, and cardiac output. We have added the detailed analyses of cardiac mechanics derived from c-LSFM imaging in Table S1.

In addition, we applied retrospective synchronization algorithms and resolution-enhancement post-processing to the raw c-LSFM datasets to enhance spatial and temporal resolution. Our c-LSFM system is capable of capturing the beating embryonic zebrafish (3-D space + time) and meso-scale neonate mouse hearts. While the current cardiac cycle synchronization and spatial resolution enhancement algorithms were separately implemented to the beating and isolated hearts, we anticipate further resolution enhancement in 4-D images by fusion of these two algorithms in future investigations.

Unlike the transparent zebrafish embryos, light-sheet imaging of adult zebrafish and neonatal mouse hearts entails significant light scattering due to large size and non-homogeneous samples. The current approach to visualize completely opaque hearts is to perform cryostat sectioning, followed by confocal scanning which necessitates manual and precise sectioning of the samples. The sectioned slices need to be placed on microscopy glass slides for individual imaging over several hours of neonatal mouse samples, and the individually acquired images need to be ‘stitched’ together. In this context, the use of confocal microscopy is theoretically feasible to generate 3-D and high-resolution architecture; however, the sample preparation, acquisition time, and data processing warrant a rapid and multi-scale light-sheet strategy for both living embryos and large-sized samples. Without sectioning mid-size samples such as the 120 dpf adult zebrafish heart, c-LSFM provides superior axial resolution on visualizing the cardiac trabecular network that is not able to be clearly discerned by confocal axially (Fig. S6). Besides the advantage of better axial resolution for imaging large samples, c-LSFM further shows outstanding acquisition speed advantages of plane illumination over confocal’s point scan. In our control experiment, the acquisition rate of c-LSFM is ~209 mega pixels per second (8 s for 2048*2048*400 volumetric image) *versus* 0.19 mega pixels per second of confocal (1800 s for 1024*1024*322 volumetric image). Along with the high scanning speed, the c-LSFM also has high fluorescence detection efficiency which is over 90%, due to its wide-field detection. In contrast, confocal blocks most of the excited fluorescence for out-of-focus planes (95–97% under 1 Airy unit) and thus has a very poor detection efficiency as well as higher rate of photo-bleaching.

We have further minimized optical scattering which commonly occurs in myocardial imaging. In addition to applying tissue clearing, we performed rapid plane-by-plane scanning to attenuate photo-bleaching. Following pre-processing and re-construction with both image analysis software and our post-processing algorithms, the acquired series of virtual sections (as opposed to the manually sectioned heart slices by the confocal approach) have allowed for precise 3-D re-construction of the cardiac structures. In particular, internal anatomic cardiac structures, such as valves and trabeculation, are clearly visualized. Similar to the fluorescence microscopy techniques, sub-cellular organelles of interest may be labeled prior to sacrificing the animals (e.g. transgenic zebrafish lines with green fluorescent protein-labeled *cmlc*) or may be targeted by fluorophore-labeled antibodies to specific epitopes. Intrinsically occurring auto-fluorescence from elastin and/or collagen as well as NADPH (nicotinamide adenine dinucleotide phosphate) in cardiomyocytes may bypass the need for fluorescent labeling. Despite optical clearing with BABB, streaking artifacts can remain from local absorption or scattering of the non-homogeneous sample (Fig. 5a). The streaking artifacts can be eliminated by incorporating bilateral even plane illumination to engender a thinner pivoting light sheet^24^. Thus, our c-LSFM is capable of performing optical sectioning in an intact heart for 3-D visualization and analysis of a structure of interest from various views.

Adaptive optics may be considered to enhance our c-LSFM system. Adaptive optics was first derived from Astronomy to rectify the light paths that are scattered by the atmosphere into a clear image, and has been widely used in biological imaging for recovering the signals from severely scattered tissues. Several techniques, such as Optical Phase Conjugation, Digital Optical Phase Conjugation, Wavefront Reversion, have been invented to refocus the scattered light for deep tissue penetration. The adaptive optics is designed to address the turbid medium in which the vast majority of components are beyond the ballistic regime. In our work, the zebrafish embryo heart is highly transparent without significant scattering observed. Both the adult zebrafish and neonatal mouse hearts are opaque. To visualize ultra-structures such as valves and trabecular formation, we chemically cleared the heart samples, rendering them transparent for laser penetration into the entire tissue and for fluorescent signal collection without scattering. Thus, our focus is to establish the application of LSFM for visualizing both anatomy and function/physiology of transparent and scattering-free cardiac samples with high spatial-temporal and depth resolution.

Using the c-LSFM strategy, we observed that doxorubicin treatment, an anthracycline class of chemotherapeutic agent, induced an enlarged ventricular cavity but cardiac hypertrabeculation in an attempt to provide oxygenation and nutrition to the myocardium for maintenance of cardiac contractile function^21^. Besides the adult zebrafish hearts, our c-LSFM has further provided the axial resolution to elucidate the 3-D helical architecture of cardiomyocytes in association with the programmed perinatal changes of cardiac mechanics for coordinated cardiac contractile function^25^. Cardiomyocyte architecture has been visualized by 3-D fiber tracking and quantified by measuring helix angle via diffusion-weighted magnetic resonance imaging (MRI) in fetal, neonatal, and adult pig hearts^26^. The helical architecture of cardiomyocytes was observed as early as the mid-gestational period, and postnatal changes of cardiomyocyte architecture were observed from postnatal day 1 to 14 in the septum and right ventricular free wall^26^. The transmural gradient of cardiomyocyte orientation undergoes progressive changes during cardiac development in response to mechanical workload^27^. Superior to the contrast-enhanced microcomputed tomography (micro-CT) imaging^7^, our c-LSFM elucidated the distinct orientation of cardiomyocyte fibers, muscular ridges and trabeculation in the left atrial appendage, as well as papillary muscle in the left ventricle (Fig. 6)^28^. Thus, a c-LSFM strategy opens a new avenue to unravel functional and structural phenotypes in genetically engineered mouse models otherwise challenging with micro-CT or fetal echocardiography^29^, to elucidate lineage tracing of cardiomyocyte blasts^30^ and patterns of mesenchymal-endothelial transition for neovascularization^31^.

## Methods

### Ethics statement

Zebrafish and mice experimentation in this study was carried out in accordance with animal protocols approved by UCLA Institutional Animal Care and Use Committee (IACUC) protocols (ARC#: 2015-055 and 2012-039).

### Preparation of the Transgenic and Mutant Zebrafish Lines

In compliance with the UCLA IACUC protocols, transgenic *Tg*(*cmlc2:gfp*) lines were raised in the UCLA Zebrafish Core facility. *Cmlc2* is expressed with gfp, and is cardiomyocyte-specific^32^. To maintain optical clearance of the embryos, the medium was supplemented with 0.003% phenylthiourea (PTU) to suppress pigmentation at 20 hpf 33. *Tg*(*cmlc2:gfp*) embryos were anesthetized in 0.05% tricaine^34^,^35^ and immersed in 0.5% low-melt agarose at 37^o^ C from 50 to 100 hpf. This procedure allowed for imaging myocardial movement prior to agarose solidification. The embryos immersed in low-melt agarose were transferred to a fluorinated ethylene propylene (FEP) tube (Refractive Index (RI): ~1.33) to provide optical clarity for fluorescence detection. The FEP tube was immersed in a water chamber (water RI: ~1.33) connected to the LSFM system. The samples were allowed to rotate in the x-y-z directions via an automated device.

### Doxorubicin (DOX) Treatment to Adult Zebrafish Hearts

Transgenic zebrafish *Tg(cmlc2:GFP)* lines with gpf-labeled cardiac myosin light chain were used to allow visualization of the 3-D cardiac structural reorganization in response to DOX, an anthracycline agent of chemotherapy. The aforementioned optical clearing technique enabled the gfp signal to remain unquenched. Adult fish following DOX injection were imaged by LSFM at 30 days following injection. Images were analyzed using ImageJ and Amira softwares, allowing delineation of the volumes of interest.

### Murine heart preparation

In compliance with the UCLA IACUC protocols, *αMHC*^Cre^ (B6.FVB-*Tg*(*Myh6-cre*)2182Mds/J) and *TdTomato* (B6;129S6-*Gt(ROSA)26Sor*^*tm9(CAG-tdTomato)Hze*^/J) mice were obtained from the Jackson laboratory (ME, USA). To readily identify cardiomyocytes based on intrinsically expressed fluorescent proteins, we generated a double transgenic mouse model (*αMHC*^*Cre*^*;TdTomato*) in which cardiomyocytes were indelibly marked by TdTomato. Hearts were harvested from one to three days old *αMHC*^*Cre*^*;TdTomato* mice. Prior to extraction, hearts were stopped in diastole by injection of KCl (3 M) followed by washing with 1X PBS. The tissue was subsequently fixed in 4% paraformaldehyde for 2 hours, followed by three washes with 1X PBS.

### Optical Clearing of Myocardium

The agarose gel containing the heart samples (adult zebrafish heart or neo-natal mouse hearts) was dehydrated by immersing the gel into 40, 60, 80 and 100% ethanol at room temperature, for 10 to 30 minutes sequentially. Next, the agarose gel with the samples was immersed into a BABB solution (1:2 benzyl alcohol:benzyl benzoate) for 30 to 120 minutes, to remove the lipid components from the cell membranes and replace them with BABB solution. Then the refractive index of chemically treated tissue was perfectly matched with that of BABB (RI ~1.51). The optical clearing greatly reduced scattering from thick cardiac tissues and enabled light transmission through the entire transparent heart.

### 4-D Imaging of Embryonic Zebrafish Hearts

We incorporated post-computation with c-LSFM to study the cardiac mechanics of live embryonic zebrafish hearts. Living *Tg*(*cmlc:gfp*) fish embryos were selected for 4-D visualization of *cmlc*-labeled myocardium. As the light-sheet sectioned a thin layer of the beating heart at a certain z depth, the sCMOS camera (Hamamatsu ORCA flash 4.0) continuously recorded the dynamic plane images of this layer (depth) for 4 to 5 cardiac cycles at a high frame rate of ~ 100 fps. Every 300 frames were acquired from each z layer in response to the relatively fast heart rate (~2 bps) of zebrafish embryos. We reiterated this process for each z layer till the light-sheet scanned through the entire heart^36^. A retrospective synchronization algorithm was applied to the non-gated LSFM dataset, followed by computational synchronization of the cardiac cycles at different z layers^10^,^36^. Finally, we sequentially reconstructed the multiple 3-D structures from systole to diastole during a cardiac cycle to generate a “3-D beating heart” in both spatial and time domains (Figs S5 and S7).

There are four sequentially executed image processing steps to reconstruct the zebrafish beating heart. These are period determination, relative shift determination, absolute shift determination and post-processing. First, period determination is designed to estimate an accurate heart rate. In short, we iterate through a set of estimated period hypotheses, and back-project all samples into the first period with respect to the hypotheses. By comparing the samples at the same spatial location, we evaluate each of the period hypotheses. The best hypotheses will be selected accordingly. Second, relative shift determination aimed at aligning the starting sample of each individual image sequences. Starting from a number of relative shift hypotheses, we adopted a quadratic cost function to measure the alignment. By maximizing the alignment, we select the best possible relative shift for each image sequence with respect to the other sequences. Third, absolute shift determination targeted to obtain the absolute shift of each individual image sequence with respect to the first sequence. We use the relative shift result, and calculate a weighted averaged shift for each slice respectively. Finally, we post-process the images by resampling each image sequence with respect to period, truncate image sequence to align the starting sample, and remove noise.

### Confocal Microscopy

Zeiss LSM 5 PASCAL was used to compare the image resolution and acquisition speed against c-LSFM. After clearing 120dpf zebrafish heart, we placed it on a coverslip and imaged with a 10X/0.3 air objective lens.

### Quantification of Cardiac Mechanics

To assess changes in ventricular function during cardiac development, volumetric dimension throughout the cardiac cycle was acquired by LSFM at 100 hpf using a sCMOS camera. Captured images were used for segmentation to create a 2-D moving boundary with 600 nodes^37^. The nodes were guided to provide cardiac wall motion as previously described^37^. Matlab (Mathworks, Natick, MA, USA) was used to calculate the global longitudinal strain rates based on changes in displacement 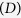 between two time frames^38^.


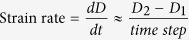


where *D*_*1*_ denotes the early stage and *D*_*2*_ the later stage at a time-step of 0.05 seconds. Based on LSFM images coupled with non-gated 4-D synchronization computational algorithm^10^,^36^, changes in end systolic (ESV) and end diastolic volume (EDV) were determined by the Amira imaging software (FEI software, Hillsboro, OR)^38^.

## Additional Information

**How to cite this article**: Fei, P. *et al.* Cardiac Light-Sheet Fluorescent Microscopy for Multi-Scale and Rapid Imaging of Architecture and Function. *Sci. Rep.* **6**, 22489; doi: 10.1038/srep22489 (2016).

## Supplementary Material

Supplementary Information

Supplementary Video S1

Supplementary Video S2

Supplementary Video S3

Supplementary Video S4

Supplementary Video S5

Supplementary Video S6
