# Supplementary material for: Cardiac Light-Sheet Fluorescent Microscopy for Multi-Scale and Rapid Imaging of Architecture and Function
Source: Sci Rep. 2016 Mar 3;6:22489. doi: 10.1038/srep22489 (PMC4776137; doi:10.1038/srep22489)
Supplement: Supplementary Information [file srep22489-s1.pdf]

Supplementary Information

Cardiac Light-Sheet Fluorescent Microscopy for Multi-Scale and Rapid Imaging of Architecture and Function

Peng Fei[1,2]*, Juhyun Lee[3]*, René R. Sevag Packard[3,4,5*], Konstantina-Ioanna Sereti[5], Hao Xu[6], Jianguo Ma[5], Yichen Ding[5], Hanul Kang[5,7], Harrison Chen[3], Kevin Sung[3], Rajan Kulkarni[5], Reza Ardehali[5], C.-C. Jay Kuo[6], Xiaolei Xu[8], Chih-Ming Ho[2], Tzung K. Hsiai[,3,4,5,7]

[1]School of Optical and Electronic Information, Huazhong University of Science and Technology, Wuhan, China, [2]Department of Mechanical & Aerospace Engineering, University of California, Los Angeles (UCLA), Los Angeles, CA, [3]Department of Bioengineering, UCLA, Los Angeles, CA, [4]Department of Molecular, Cellular and Integrative Physiology, UCLA, Los Angeles, CA, [5]Division of Cardiology, Department of Medicine, UCLA, Los Angeles, CA, [6]Department of Electrical Engineering, University of Southern California, Los Angeles, CA, [7]Division of Cardiology, Veterans Affairs Greater Los Angeles Healthcare System, Los Angeles, CA, [8]Department of Biochemistry and Molecular Biology, Mayo Clinic, Rochester, MN,

*These authors contributed equally

Corresponding Author:
Tzung K. Hsiai, MD, PhD, Departments of Medicine and Bioengineering, University of California, Los Angeles, Los Angeles, CA. Email: THsiai@mednet.ucla.edu. Phone: 310-268-3839. Fax: 310-268-4288

Supplementary materials:

**Fig. S1**	The schematic diagram of c-LSFM modality
**Fig. S2**	4-D zebrafish beating heart reconstruction methods
**Fig. S3**	Post-image processing of adult zebrafish heart with resolution enhancement by different deconvolution techniques.
**Fig. S4**	The P1 neonate mouse hearts before and after BABB clearing
**Fig. S5**	Synchronization comparison before and after.
**Fig. S6**	The imaging comparison between c-LSFM and confocal microscope using 120 dpf zebrafish hearts.
**Fig. S7**	Comparison of 4-D synchronized images with a combination of 3 different parameters
**Table S1**	Analysis of cardiac mechanics in 4dpf zebrafish from 4-D *in vivo* imaging
**Video S1**	The high speed, high contrast imaging of live embryonic zebrafish heart
**Video S2**	Cardiac cycle before temporal interpolation
**Video S3**	Cardiac cycle after temporal interpolation
**Video S4**	The 4D reconstruction of live embryonic zebrafish heart
**Video S5**	The image acquisition process of c-LSFM
**Video S6**	The high resolution reconstruction of digital mouse heart using Amira

(a) (b)

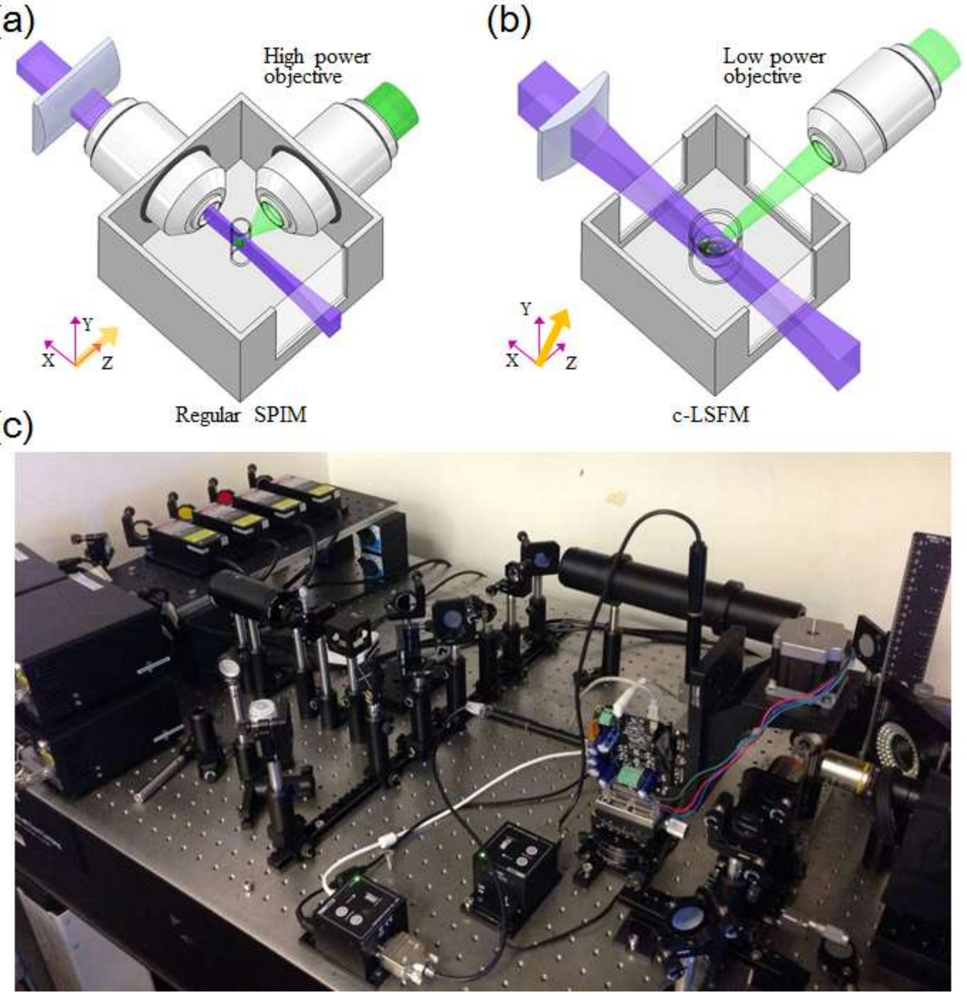

(c)

Figure S1. The schematic diagram of cardiac-LSFM (c-LSFM) modality. **(a)** The regular SPIM seals two high power, water immersion lenses into a water chamber, aligning them nearby the small sample, for both illumination and detection. **(b)** In contrast, the c-LSFM system uses low power, long working distance, air objectives with ample room between the sample and the front pupil of objective. **(c)** The photograph of the c-LSFM system shows that on one hand, the large sizes of the cardiac structures necessitate a large working space for scanning of entire samples as well as convenience of operation. On the other hand, the involvement of the resolution enhancement technique substantially decouples the need of high N.A., water dipping objectives from the high resolution, large space-bandwidth product.

Furthermore, eliminating the sealing of the objectives into a water chamber additionally benefits the frequent changes in immersing mediums for different samples. Therefore, the c-LSFM modality we specially designed is very efficient in performing trans-scale cardiac light sheet imaging conveniently.

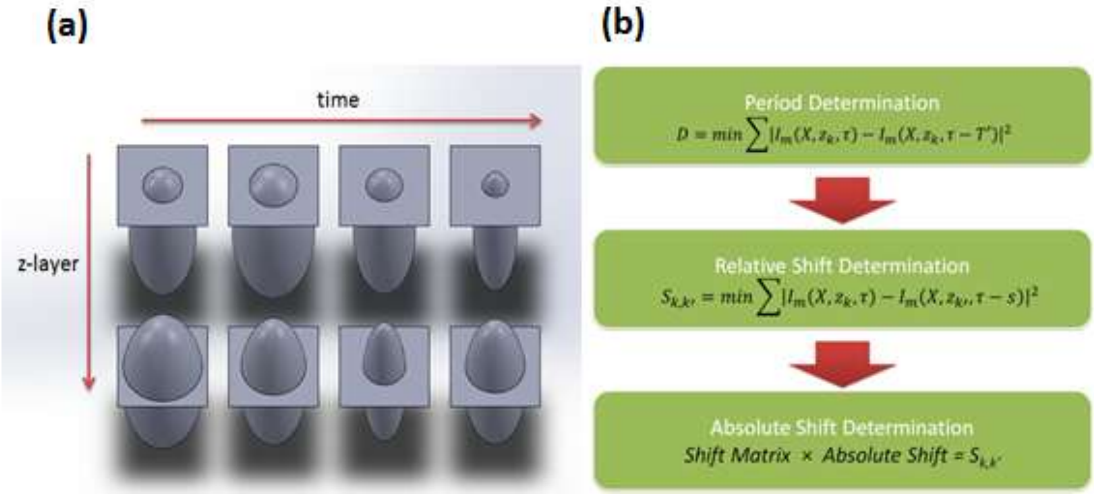

Figure S2. 4-D zebrafish beating heart reconstruction methods. **(a)** When scanning in a single layer, cardiac contraction is time-dependent. While moving in the z-axis to scan the following z layer, we captured time-dependent contraction images from each layer from top to bottom of the heart. **(b)** For proper synchronization, heartbeat period was determined by frame. We iterated through a set of hypothesized periods and back-projected all samples into the first period with respect to the hypotheses. After back-projection, we compared samples at a same spatial location but from different periods and evaluated each of the period hypotheses. The best hypotheses were selected accordingly. Relative shift determination aimed at aligning the starting sample of each individual image sequence. The heart may not be in the same contraction state at the beginning of all sequences when we start taking images at each z layer. Starting from a number of relative shift hypotheses, we adopted a quadratic cost function to measure the alignment. The cost function is calculated via measuring the similarity between two hypothetically aligned images from two adjacent image sequences with respect to the relative shift hypotheses. By maximizing the alignment, we select the best possible relative shift hypothesis for

each image sequence with respect to the other sequences. Absolute shift determination targeted to obtain the absolute shift of each individual image sequence with respect to the first sequence. In the previous step, relative shift between any close-by image sequences are obtained. We recursively calculate relative shift between the current image sequence and an early sequence until obtaining the relative shift with respect to the first image sequence. The above process is applied to every image sequence, and all such processes can be compactly implemented by one matrix multiplication.

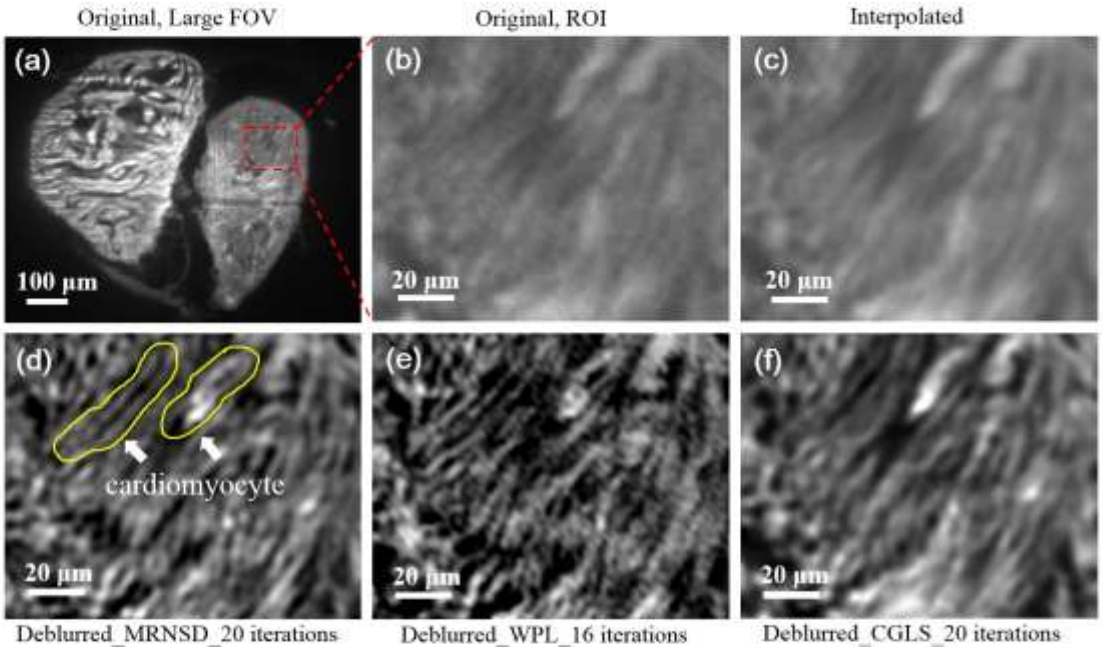

Fig. S3. Post-image processing of adult zebrafish heart with resolution enhancement by different deconvolution techniques. (a) Zebrafish atrium and ventricle are visualized on one section of raw image. (b) The individual cardiomyocytes were unresolvable from the selected region-of-interest from the atrium due to the optics blurring and under-sampling by the camera. (c) The image was first scaled up with 3X b-spline interpolation to partially recover the information loss from incomplete sampling. (d), (e) and (f) illustrate the interpolated images deblurred by iterative MRNSD, WPL and CGLS deconvolution, respectively. The WPL algorithm generates most effective deblurring to recover sharp and high frequency signals. However, it also generates image discontinuity, likely due to the application of wiener filter. The CGLS algorithm appears to be mild, generating the least degree of deblurring. Of the three resolution enhancement algorithms, the MRNSD method provides optimal trade-off between the resolution enhancement and information preservation.

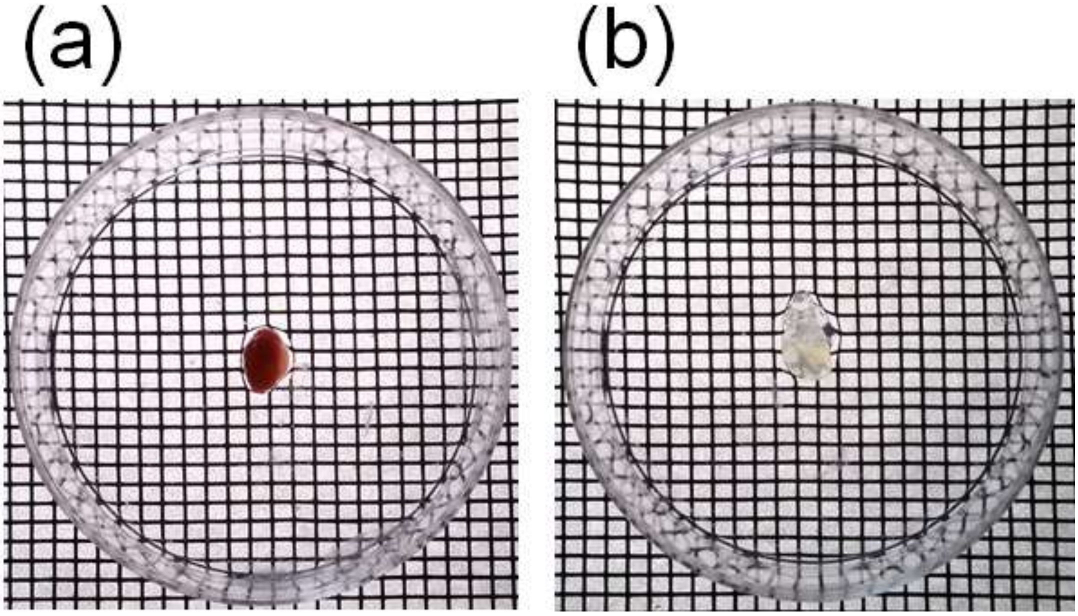

Fig. S4. The day 1 neonatal mouse hearts before and after BABB clearing. **(a)** Photograph of the raw isolated hearts. **(b)** Photograph of the cleared heats treated by a rapid BABB clearing. BABB chemical clearing of the tissue is fast and potent. We optically cleared the day 1 neonatal mouse heart with 2 hours serial ethanol dehydration followed by 2 hours benzyl alcohol-benzyl benzoate clearing. Compared to the raw hearts that were completely opaque before clearing **(a)**, the treated hearts showed significantly reduced scattering and became highly translucent on a scale board.

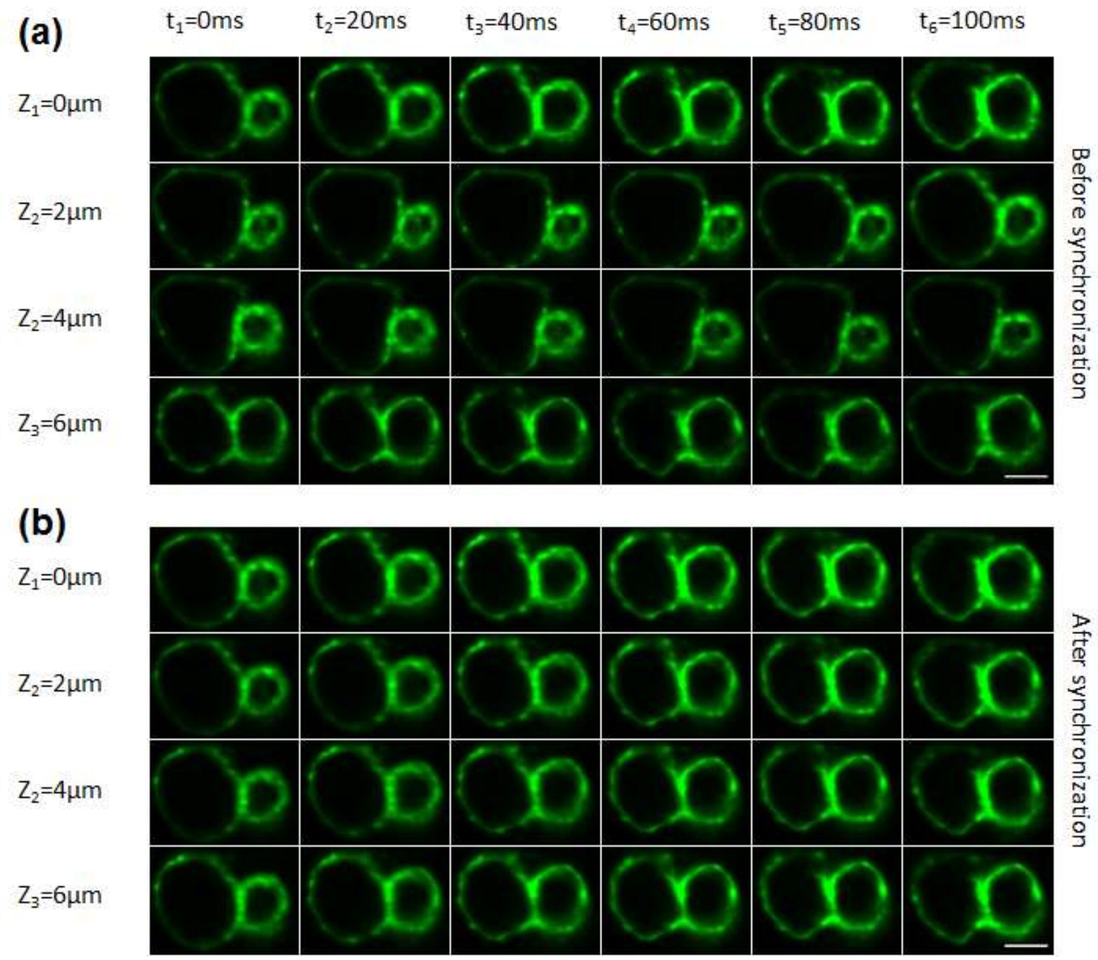

Figure S5. Comparison between prior to and post 4-D synchronization algorithm. (a) Before synchronization, zebrafish cardiac contractions at different Z positions were not in the same stage. (b) After synchronization, all Z positions were synchronized in the same cardiac contraction stage. Therefore, stacked images for 3-D reconstruction at certain time points were obtained and provided volume information. Scale bar = 50μm.

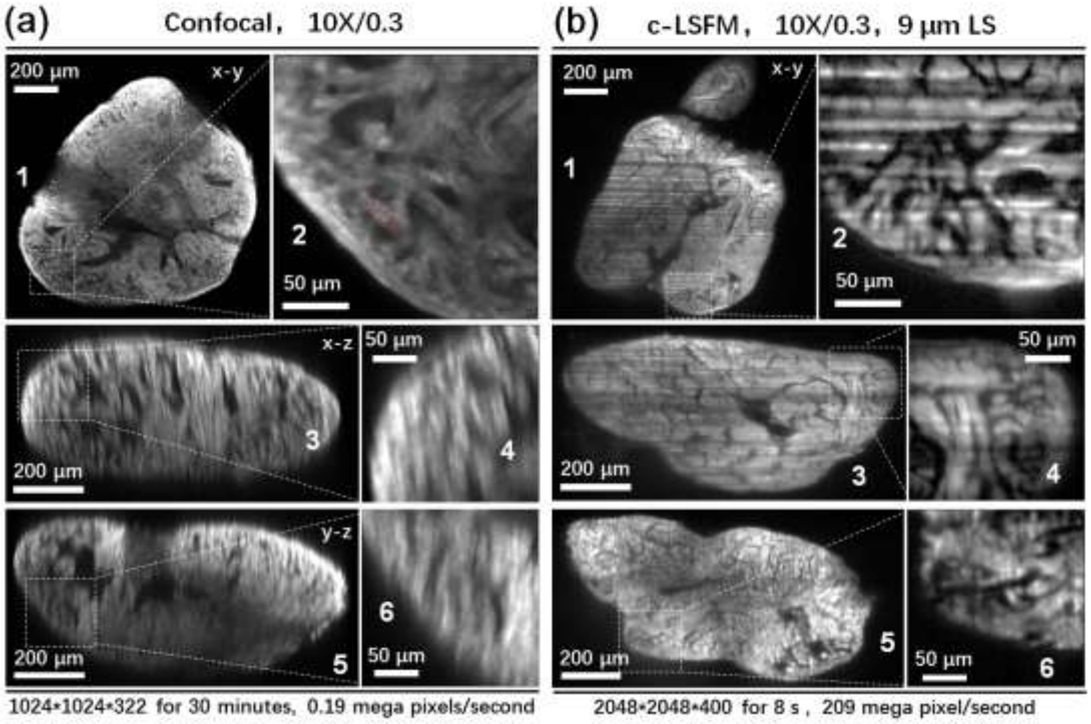

Fig. S6. Imaging comparison between c-LSFM and confocal microscopy using 120 dpf zebrafish hearts. (a) The original x-y plane image, reconstructed x-z and y-z plane images obtained from confocal data. (b) The original x-y plane image, reconstructed x-z and y-z plane images obtained from c-LSFM data. The zoomed-in images shown in the right columns (2, 4, 6) indicate the lateral and axial resolving powers of confocal and c-LSFM.

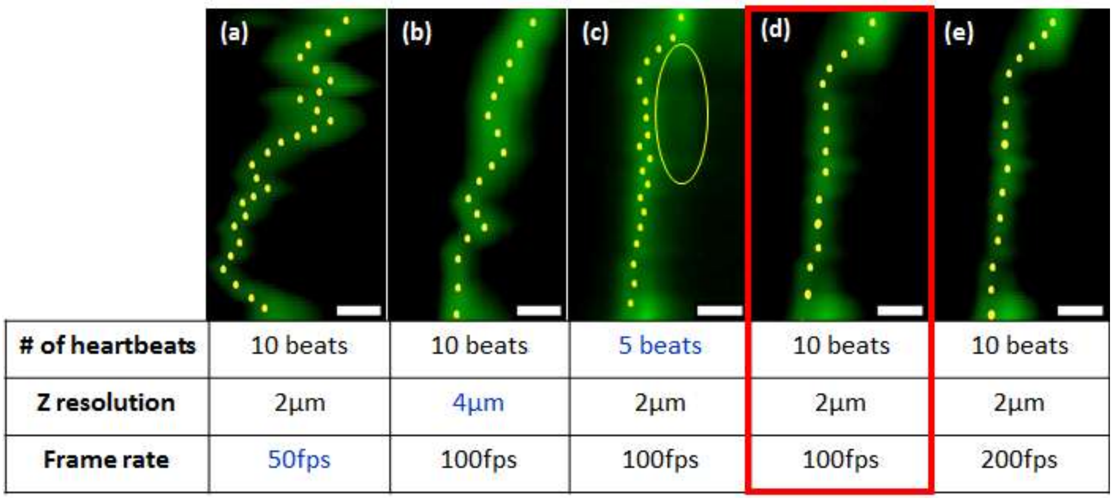

**# of heartbeats**	10 beats	10 beats	5 beats	10 beats	10 beats
**Z resolution**	2μm	4μm	2μm	2μm	2μm
**Frame rate**	50fps	100fps	100fps	100fps	200fps

Figure S7. Comparison of 4-D synchronized images with a combination of 3 different parameters. (a & b) Both low frame rate and low z resolution were inadequately synchronized. Both images demonstrated a crinkled pattern in the cardiac wall. (c) Reducing the capturing number to 5 heartbeats (cardiac cycles) revealed similar synchronization with capturing 10 beats. However, negligible artifact appeared behind the wall (yellow circle). (d & e) Increasing the frame rate to 200fps revealed identical image quality to that of 100fps. Therefore, we selected (d) as the optimal combination for 4-D synchronized imaging parameters. Scale bar = 10μm.

Table S1. Analysis of cardiac mechanics in 4dpf zebrafish from 4-D in vivo imaging.

End-Diastolic Volume ($10^5 \mu m^3$)	5.5$\pm$0.6
End-Systolic Volume ($10^5 \mu m^3$)	1.4$\pm$0.5
Stroke Volume ($10^5 \mu m^3$/beat)	4.1$\pm$0.5
Ejection Fraction (%)	74.5$\pm$5.6
Heart Rate (bpm)	164$\pm$20
Cardiac Output ($10^5 \mu m^3$/min)	672.4$\pm$15

Video S1 shows the high speed, high contrast imaging of a live embryonic zebrafish heart. The fast interaction of blood flow (ds-red) and beating cardiac muscle (GFP) was captured and synchronized.

Video S2 shows the beating heart of a live embryonic zebrafish before temporal interpolation.

Video S3 shows the beating heart of a live embryonic zebrafish after temporal interpolation.

Video S4 shows the 4-D reconstruction of a live embryonic zebrafish heart.

Video S5 shows the acquisition process of day 1 neonatal mouse heart using c-LSFM. The frame rate was as fast as 20 fps under full resolution, which enabled 3D scanning of an entire heart in around 30 seconds.

Video S6 shows the high resolution, digital neonatal mouse heart reconstructed using Amira. We can easily access an area of interest with a cellular level resolution.
